# A Systematic Review of Cystic Fibrosis in Children: Can Non-Medical Therapy Options Lead to a Better Mental Health Outcome?

**DOI:** 10.7759/cureus.37218

**Published:** 2023-04-06

**Authors:** Natalie A Gonzalez, Sana M Dayo, Umaima Fatima, Aaiyat Sheikh, Chaitanya S Puvvada, Faiza H Soomro, Hafsa A Osman, Merna Haridi, Safeera Khan

**Affiliations:** 1 Pediatrics, California Institute of Behavioral Neurosciences & Psychology, Fairfield, USA; 2 Public Health Sciences, Liaquat University of Medical and Health Sciences, Jamshoro, PAK; 3 Obstetrics and Gynaecology, California Institute of Behavioral Neurosciences & Psychology, Fairfield, USA; 4 Internal Medicine, California Institute of Behavioral Neurosciences & Psychology, Fairfield, USA; 5 Internal Medicine, Era's Lucknow Medical College and Hospital, Lucknow, IND; 6 General Surgery, California Institute of Behavioral Neurosciences & Psychology, Fairfield, USA; 7 General Surgery, Gayatri Vidya Parishad Institute of Health Care and Medical Technology, Visakhapatnam, IND; 8 General Surgery, Ninewells Hospital, NHS Tayside, Dundee, GBR; 9 Medical Education, Saint Martinus University, Willemstad, CUW; 10 Research, California Institute of Behavioral Neurosciences & Psychology, Fairfield, USA

**Keywords:** alternative treatments, complementary therapies, depression, mental health, pediatric disease, cystic fibrosis

## Abstract

Cystic fibrosis (CF) is a chronic disorder that begins at an early age, so it is crucial to be aware of the physical and emotional burden placed on individuals suffering from it and their families. It significantly impacts an individual's life; therefore, it is essential to acknowledge the effects of the disease on physical and mental health. Our systematic review aims to highlight the areas of life affected by cystic fibrosis and evaluate various non-medical treatment options that may support the mental health of CF patients. We selected PubMed, Google Scholar, and MEDLINE (Medical Literature Analysis and Retrieval System Online) as our databases. We initially found 146,095 articles and narrowed the number of articles down using filters, exclusion and inclusion criteria, and various combinations of Medical Subheadings (MeSH) and key terms. We decided to use a final count of nine articles for our systematic review. The studies we included highlighted the negative impact of cystic fibrosis on mental health, like depression and anxiety, as well as on sleep, physical health, and overall quality of life. Several non-medical interventions, such as logotherapy, psychological interventions, complementary and alternative medicine, and many more, have been shown to enhance the mental health of many participants. Studies suggested that such therapy options may greatly benefit individuals with cystic fibrosis and their current treatment plan. This review indicates that non-medical therapy options can enhance the mental health of individuals suffering from cystic fibrosis and that it is crucial to bring more attention to preventing and treating mental health issues in cystic fibrosis patients. However, as current data is limited, more research with a larger number of participants over an extended period of time is necessary to better evaluate the efficacy of non-medical interventions on mental health.

## Introduction and background

Globally, between 70,000 and 100,000 people suffer from the genetic disorder cystic fibrosis [1]. Over the years, the survival rate drastically increased, with a median age of almost 50 years [2]. Although this marks a significant improvement for individuals suffering from this disease, as it is a chronic one, it has a remarkable impact on not only physical but also mental health and many other aspects of an individual's life. Mental health issues such as anxiety and depression are common comorbidities in patients and their parents [1]. Cystic fibrosis (CF) patients who suffer from a depressive disorder are less adherent to medical treatments and, therefore, may have a worse health outcome [3]. Also, the financial aspect has to be taken into consideration. A study assessed the healthcare costs in individuals with cystic fibrosis with or without depression, and results showed that the healthcare costs for CF individuals who suffered from depression were annualized at $280,000 on average, as compared to $60,116 on average for individuals who did not suffer from a mental health disorder [3].

Individuals with cystic fibrosis require lifelong treatment of their symptoms, as there is no cure for this disease. A high number of various mutations of the cystic fibrosis transmembrane conductance regulator (CFTR) gene are responsible for many different phenotypes in this disease. If the function of CFTR is impaired or absent, viscous mucus will build up in the lungs and other organ systems, such as the gastrointestinal tract. The buildup of mucous in the pancreas can cause its destruction, leading to exogenous pancreas insufficiency. CF patients then suffer from poor nutrition and, eventually, CF-related diabetes (CFRD). Individuals suffer from frequent infections, especially pulmonary ones, which lead to progressive lung function decline. Despite affecting other organs, respiratory failure is still the primary cause of death [4].

Considering these tremendous effects on physical health, it is no surprise that CF patients struggle more often with their mental health. Furthermore, they are less likely to adhere to their medication and, therefore, have a higher risk of admission to the hospital [5]. Not paying attention to the mental health of CF patients can negatively impact their health-related quality of life (HRQOL) [6]. Evidence has also suggested that symptoms of mental health issues such as anxiety and depression are also prevalent in the parents of CF patients. One study reported that more than 49%-66% of caregivers suffered from mental health issues, with many having moderate-to-severe symptoms [7].

Various non-medical treatment options exist with the intent to support the mental health of cystic fibrosis patients. Many patients use complementary and alternative medicine (CAM), with prayer being the most common. Individuals subjectively reported benefits from CAM [5]. Other forms of CAM, including taking natural supplements, using scented candles or aromatherapy, meditation, and healing touch, also showed to help CF patients [6]. CF patients and, if needed, their caregivers may also benefit from numerous psychological interventions such as behavioral therapy, hypnosis, biofeedback, and physical activity [8]. Helping affected individuals find new meaning in their lives and supporting them as they process the physical and mental challenges they face is crucial. For this matter, using logotherapy successfully improved the mental health of many CF patients in a study [9].

As CF is a rare disease, only limited data exist regarding the effect of mental health issues on physical health and further appropriate treatment for them. We decided to conduct this systematic review to investigate current non-medical treatment options and their impact on emotional well-being in CF patients. Our definition of the term non-medical therapies includes e-health (electronic health) interventions, complementary and alternative medicine, psychological interventions such as behavioral therapy, and logotherapy. The goal of our systematic review is to focus on existing data on this crucial topic and raise awareness of its importance. We will address current non-medical treatments and evaluate if they can efficiently support the mental health of CF patients, increase their quality of life, and maybe even reduce physical symptoms.

## Review

Methods 

**Figure 1:**
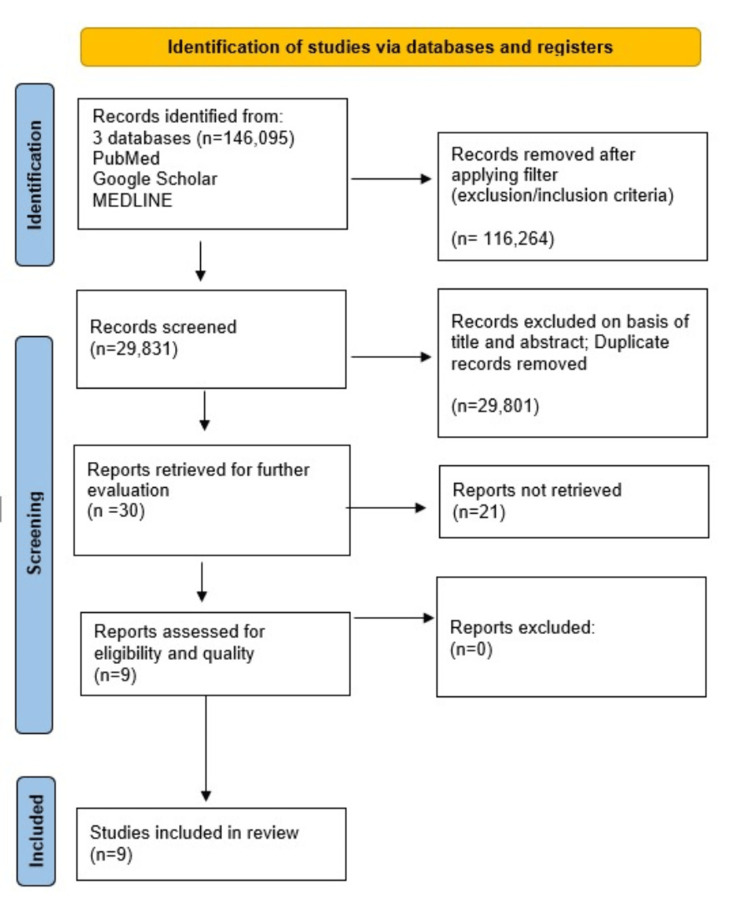
PRISMA Flowchart of Selected Articles. PRISMA: Preferred Reporting Items for Systematic Reviews and Meta-Analyses; MEDLINE: Medical Literature Analysis and Retrieval System Online.

We followed the Preferred Reporting Items for Systematic Reviews and Meta-Analyses (PRISMA) Guidelines 2020 for this systematic review [10]. To further illustrate the selected articles used, the PRISMA flowchart, as shown in Figure 1, will show the process in more detail [10]. 

Search Strategy

We used three databases for this systematic review: PubMed, Google Scholar, and the Medical Literature Analysis and Retrieval System Online (MEDLINE). We created three Medical Subject Heading (MeSH) concepts. We searched for articles with the different MeSH combinations "Cystic fibrosis AND mental health," "Cystic fibrosis AND Alternative therapy OR Complementary Therapies," and "Cystic fibrosis AND mental health AND Alternative therapy OR Complementary Therapies." We decided on our key terms and used different combinations for each database. The key terms we used were "Cystic fibrosis," "Children," "Mental Health," "Depression," "Complementary therapies," and finally, "Alternative treatments." As we came up with a high number of papers, we applied our inclusion and exclusion criteria to narrow down the number of articles. We sorted out articles based on the title and the abstract and applied our quality check to the remaining papers that met our criteria. Finally, nine papers satisfied our expectations.

Inclusion Criteria

We used articles in the English language from 2002 till September 21, 2022, the final day of our data search. Further, we included children of all ages and young adults from zero to 18 years old. The final papers we chose were all open access papers.

Exclusion Criteria

We did not use articles in another language than English or published before 2002. We excluded articles that focused only on adults rather than on children. We did not use articles that were not available for free.

Results

We found 146,095 articles within our three selected databases: PubMed, Google Scholar, and MEDLINE. We applied exclusion and inclusion criteria and were able to remove 116,264 articles. We analyzed the remaining 29,831 papers and decided not to use 29,801 papers because they were duplicates or because of their titles and abstracts. We evaluated the remaining 30 papers and decided to remove 21 more as their content did not satisfy our expectations. We applied our quality check on the remaining nine papers, which all selected articles met. Finally, we included all nine articles in our final systematic review. See Table 1 for an in-depth description of the articles we decided to use.

**Table 1:** Relevant studies exploring mental health-related issues and the effectiveness of non-medical therapies in children with cystic fibrosis. HADS: Hospital anxiety and depression scale; HRQOL: Health-related quality of life; UK: United Kingdom; USA: United States of America; CAM: Complementary and alternative medicine; GHQ: General health questionnaire; QoL: Quality of life; E-health: Electronic health; CF: Cystic fibrosis.

Study	Year of Publication	Location	Type of Study	Total Patient Population	Outcome
Cronly et al. [6]	2019	Ireland	Cross-sectional Study	174 participants between 14 and 18 years	The study used the HADS score and the Cystic Fibrosis Questionnaire-Revised. It showed that poor mental health negatively influenced the quality of life. Paying more attention to mental health led to better HRQOL.
Francis et al. [11]	2020	Australia	Mixed Methods Study	22 participants between 12 and 17 years old	After using the smartphone application CyFi Space for six weeks, most participants reported that it was easy to use. They would further recommend it to their friends as they enjoyed using it. It also helped them to connect with other children affected by cystic fibrosis. Participants reported a moderate acceptance of the application and further suggestions for improvements.
Goldbeck et al. [8]	2014	UK, USA, Germany	Systematic Review	A total of 16 studies with 556 participants, between 18 months and 41 years of age	Using psychological interventions in individuals with cystic fibrosis suggests that they can improve physical and mental health. Behavioral therapy seemed to support children's nutrition and growth status for a short time. Educating patients on critical decisions like lung transplantation supported the decision-making process positively. Interventions such as biofeedback-assisted breathing positively influenced certain lung functions.
Graziano et al. [7]	2020	Italy	Cross-sectional Study	167 patients starting at age 12 and 186 parents	Most patients and their parents met the requirements for anxiety and depression, with 30% showing signs of middle-to-severe symptoms. Poor physical health, such as low pulmonary function, is correlated with a higher level of anxiety. The study suggests that mental health screening is crucial to finding individuals who require psychological treatment.
Grossoehme et al. [12]	2013	USA	Qualitative Study	Caregivers of children with cystic fibrosis between three months and thirteen years old (n=25)	19 out of 25 parents of children who suffer from cystic fibrosis integrated CAM into their children's therapy. Prayer was the most commonly used CAM, alone or within a group. Other subjectively helpful measures were using aromatherapy, lighting candles, and taking a natural oral supplement. Some parents also reported using meditation and healing touch with their children.
Nematollah et al. [9]	2021	Iran	Quasi-experimental Study	22 children between 12 and 16 years of age	The control and experimental groups answered the GHQ questionnaires before and after logotherapy sessions. The experimental group showed improvements in their mental health compared to the control group.
Oliver et al. [13]	2014	USA	Cross-sectional Study	Participants between 14 and 25 years of age (n=72)	The different questionnaires used to assess stigma and optimism showed that participants with more stigma had lower pulmonary function, less optimism, and lower QoL. They also experienced more stress. Being optimistic had a positive influence on mental health symptoms.
Thabrew et al. [14]	2018	New Zealand	Systematic Review	463 participants, children between 10 and 18 years old	Compared with the control group, there was no strong evidence that e-health interventions have a better effect on depression, anxiety, treatment acceptability, quality of life, or physical condition. Also, e-health interventions may be more challenging for children younger than 10.
Vandeleur et al. [15]	2018	Australia	Cross-sectional Study	142 participants, ages 7 to 12 years and 13 to 18 years	Children aged seven to 12 years with CF had a lower quality of sleep and mood and a lower HRQOL than the control group. Participants aged 13 to 18 years showed the same results regarding sleep quality and HRQOL. However, mood symptoms were the same as in the control groups.

Quality Check

We assessed the eligibility of our selected papers with different quality appraisal tools. We used the Assessment of Multiple Systematic Reviews (AMSTAR) criteria for all our systematic reviews and meta-analyses. Another quality appraisal tool was the Newcastle-Ottawa Tool Scale for non-randomized clinical trials. We used the Critical Appraisal Skills Programme (CASP) Checklist for qualitative studies. If we could not classify an article, we decided to use the Scale for the Assessment of Narrative Review Articles (SANRA) scale to assess the paper's quality.

Discussion

The Influence of Mental Health on the Quality of Life in Patients With Cystic Fibrosis

A cross-sectional study by Cronly et al. investigated the influence of mental health on the quality of life in patients with cystic fibrosis [6]. In this study, 174 patients between the ages of 14 and 18 participated. Participants initially filled out a questionnaire that included information about pulmonary function, especially Forced Expiratory Volume in one second (FEV1), physical appearances such as weight and height, and other sociodemographic information. This study used the Hospital Anxiety and Depression Scale (HADS) to assess mental health symptoms such as anxiety and depression. The Cystic Fibrosis Questionnaire-Revised was used to determine the quality of life [6]. The study found a negative association between the HADS and Cystic Fibrosis Questionnaire-Revised scores. About 67 of the patients included had a high anxiety score, and 20 patients reported symptoms of depression. The study concluded that poor mental health was also associated with lower health-related quality of life (HRQOL). Sociodemographic parameters did not significantly influence HRQOL [6]. One of the limitations of this study was the small sample size in the recruiting process. A possible bias could be that the questionnaires were self-reported, and there was no possibility to verify all given medical information. This study shows the urgency of prioritizing mental health as it can affect the quality of life positively or negatively. The authors suggest that additional research over a more extended time is necessary to determine more factors that affect the quality of life in patients with cystic fibrosis [6].

The cross-sectional study of Vandeleur et al. evaluated the association of emotion, quality of sleep, and health-related quality of life (HRQOL) in individuals with cystic fibrosis (CF) [15]. The study included a total of 142 participants, which consisted of children with CF between seven and 18 years and healthy controls [15]. Researchers monitored the sleep quality by overnight oximetry and with actigraphy for 14 days. To assess further information about sleep quality, such as sleepiness throughout the day, participants filled out the Pediatric Daytime Sleepiness Scale (PDSS). The Children's Depression Inventory (CDI) questionnaire and the Becks Depression Inventory for youth (BDI) questionnaire gathered detailed information about mental health. The study assessed the health-related quality of life (HRQOL) in patients with CF by using the Cystic Fibrosis Questionnaire-Revised (CFQ-R) and, in the control group, the Pediatric Quality of Life Inventory (PedsQL) [15]. The study concluded that participants with CF showed less sleep quality and were sleepier. Children with CF from the ages of seven to 12 also showed a lower mood than the control group. However, participants with CF between 13 and 18 showed the same mood as the healthy controls [15]. As poor sleep and mental health impact the HRQOL of patients with CF, it is crucial to conduct more research on how to effectively prevent and treat these disorders to help individuals with CF improve their HRQOL [15].

Effects of Mental Health on Physical Health in Patients With Cystic Fibrosis 

Graziano et al. conducted a cross-sectional study and applied the International Mental Health Guidelines regarding the annual mental health screening of CF patients [7]. This study included 167 children with CF starting at the age of 12 and 186 parents. All participants completed a self-reported screening, which a psychologist evaluated promptly [7]. If the score met the requirements for depression or anxiety, the researchers initiated appropriate interventions immediately. The study used interventions such as psychotherapy, psychoeducation, and pharmacological treatment. Participants also reported detailed information about themselves, such as Body Mass Index (BMI), history of pulmonary exacerbations, lung function, Forced Expiratory Volume in one second (FEV1), and comorbidities [7]. Most children with their parents reported experiencing some degree of depression and anxiety, with as much as 30% experiencing moderate-to-severe symptoms. The study also concluded that the severity of physical symptoms, such as lung exacerbation or hemoptysis, correlates with anxiety level and depression [7]. This study shows the critical need to implement mental health screenings in individuals with CF to guarantee the right and timely treatment [7].

A study from Oliver et al. studied the effects of stigma and being optimistic on emotional and physical well-being in patients with cystic fibrosis [13]. The study included 72 patients aged between 14 and 25 years. This study used different questionnaires. The study used the Hospital Anxiety and Depression Scale (HADS) to evaluate depression and anxiety. Researchers used Fife and Wright's Social Impact Scale (SIS) to measure the intensity of stigma. Researchers used the Life Orientation Test-Revised (LOT-R) and the Cystic Fibrosis Questionnaire-Revised (CFQ-R) to get more insights into optimism and the quality of life in patients with cystic fibrosis [13]. Researchers accessed the participants' medical records to compare the data from the questionnaires to their health data. The study focused on physical parameters such as Body Mass Index (BMI), comorbidities, pulmonary function, medication history, and records of hospitalizations [13]. Results showed that more stigma correlated with lower pulmonary function and negatively affected optimism and quality of life. Being optimistic showed a positive influence on emotional well-being and stigma [13]. A limitation of this study was that the questionnaires were self-reported. This study shows the need to pay more attention to mental health in cystic fibrosis patients to better support them and maybe even ease some of their physical symptoms [13].

Interventions to Enhance Mental Health in Cystic Fibrosis Patients

Francis et al. investigated the use of a smartphone application (app) in individuals who suffer from cystic fibrosis and whether using it can enhance their interaction with others affected by it and their mental health [11]. A total of 22 individuals between 12 and 17 years old participated. The study used the "CyFi Space" smartphone application for six weeks [11]. The app incorporated a variety of health-promoting features. Individuals were able to interact directly with each other via a chatroom. The app assessed the user's mood and shared solutions, such as going for a walk or watching a motivational video, based on their feelings [11]. Further, the app also shared contact information from appropriate counseling services if individuals required additional support [11]. A total of 85% of the participants reported that the app was easy to use. Around 60% of users reported acceptance of the app. After six weeks, participants were interviewed and shared their ideas for improvement. Participants appreciated the support provided via the app and the sharing of resources, such as information regarding counseling services. Even though the chatroom was in high demand as it made it possible to interact with other individuals, technical difficulties and the fact that they could only use this feature in an external web browser made it more challenging to use [11]. This study showed the strong desire of individuals to have an easy and safe opportunity to interact with each other and a fast way to receive guidance with this chronic disease. This app shows a promising opportunity to support individuals suffering from cystic fibrosis and help them feel more socially connected [11].

Thabrew et al. investigated in a systematic review the effectiveness of e-health (electronic health) interventions in young individuals with chronic conditions such as cystic fibrosis and if this could support their mental health, such as anxiety and depression [14]. The study evaluated five trials with 463 children between 10 and 18 years old. Researchers compared various e-health interventions with attention placebo controls or psychological placebos. Other controls were treatment, as usual, a waitlist control group, and non-psychological therapies such as medication use [14]. Participants used e-health interventions via smartphone applications (apps) or text messages. Also, studies used interactive programs via websites or web applications, virtual games, or biofeedback programs [14]. This review did not find significant changes regarding depression and anxiety in participants using e-health interventions compared to the control groups. E-health interventions did not significantly impact the quality of life or physical health in the long run [14]. The review highlighted some risk of bias as selected studies did not always blind their participants. This study concludes that the evidence for recommending e-health interventions is too low and that more development and research are necessary to provide a potential benefit for children with chronic conditions. Especially for children younger than then, e-health interventions may be more challenging to implement [14].

A qualitative study by Grossoehme et al. investigated the use of complementary and alternative medicine (CAM) in children with cystic fibrosis and their parents [12]. Researchers conducted telephone interviews with 25 parents of children with cystic fibrosis who are between three months and 13 years old. The interview evaluated if the parents used CAM with their children, its perceived effect and if they talked about using CAM with their child's treating doctor [12]. Results showed that most parents (19 of 25) used CAM with their children. The most commonly reported CAM was using prayer, either alone or within the group. Other CAMs used were healing touch, aromatherapy, candles, natural supplements, massage, and horse therapy. Parents often used a chaplain for support as well. Besides two parents, no one discussed CAM with the treating doctor of their child [12]. Parents named several reasons why they are not using CAM. One of the reasons was financial costs, and further, their child's age might not be appropriate for specific treatments such as chiropractic, difficulties in finding resources like acupuncture within the living area, and doubts that alternative therapies may not be effective. Most parents reported that their children benefited from CAM [12]. As this study's sample size was small, further research with more participants is necessary to determine the effect of CAM on children with cystic fibrosis and their parents. As participants answered the questions via a telephone interview, the chance of a recall bias also exists. All in all, the study shows that many children and their parents are subjectively benefiting from using CAM [12].

The study of Nematollah et al. investigated whether using Viktor Frankl's logotherapy positively affects the mental health of children with cystic fibrosis [9]. Twenty-two children between 12 and 16 years old were part of this study. Researchers randomly assigned the children to the experimental or control group, with both groups filling out the General Health Questionnaire (GHQ). The study used Viktor Frankl's logotherapy in the experimental group for 45 minutes weekly for nine weeks [9]. Logotherapy's primary goal is to support children's mental health, helping them navigate the difficulties of this chronic disease and find a purposeful meaning in their lives. Results showed that the mental health of the group that received logotherapy improved more than that of the control group. The experimental group's mood improved by receiving guidance through the intervention about the purpose of life, hope, and reframing a challenging situation [9]. Therefore, the study concludes that logotherapy may be an effective way to enhance the mental health of pediatric cystic fibrosis patients. As one of the limitations was the small number of participants, more research regarding the effect of logotherapy on mental health is necessary [9].

Goldbeck et al. conducted a systematic review to evaluate the benefits and effects of psychological treatments on the mental and physical health of individuals with cystic fibrosis [8]. This review includes 16 studies with 556 participants. Psychological interventions included cognitive-behavioral and psychodynamic therapies with individuals with cystic fibrosis and their families. The study also evaluated specific methods such as hypnosis, biofeedback, and other treatments, including music, dance, and physical activity [8]. Researchers assessed the effect of these therapies on mental health, life quality, social life, stress level, and physical outcomes such as lung function. Results showed that behavioral interventions, in addition to education, improved the nutrition status of patients with cystic fibrosis. Patients who need to decide for or against lung transplantation also benefit from a decision-making tool to help them with this life-changing decision. Lung function improved when participants used biofeedback-assisted breathing [8]. With various possible psychological treatment options and often multifactorial influences on mental health in cystic fibrosis patients, this review highlighted the challenge of assessing the effectiveness of psychological interventions [8]. In conclusion, many psychological interventions can potentially support the emotional and, eventually, physical health of individuals suffering from cystic fibrosis. However, more in-depth research with a larger number of participants and more specific psychological treatments is necessary to better assess the effect [8].

Limitations

Our systematic review has limitations as we only used articles in English and those published within the past 10 years. As we have focused on children as our leading population group, we do not know the data regarding adults. Another limitation of this paper is that we decided to use only articles that were available for free solely. Furthermore, as there was not an overwhelming amount of data on this topic, a more in-depth research is necessary to draw more specific conclusions.

## Conclusions

Our systematic review explored the impact of cystic fibrosis on mental health and non-medical interventions to enhance emotional well-being and further improve the quality of life in pediatric cystic fibrosis patients. The studies we included highlighted the negative impact of cystic fibrosis on emotional well-being in individuals, such as anxiety, stress, and depression, which may even negatively impact physical health. The studies showed that poor mental health led to poorer sleep quality and a lower quality of life. It also correlates with poorer physical health and can make lung function worse. A chronic disease like cystic fibrosis affects the mental health of children who suffer from it and their parents and families negatively. Therefore, screening for mental health issues as well as being proactive when it comes to treatment options is crucial. The studies we included suggested that non-medical interventions such as e-health (electronic health) interventions, complementary and alternative medicine, and psychological interventions such as behavioral therapy and logotherapy can enhance emotional well-being and improve the quality of life in pediatric cystic fibrosis patients. When patients receive the diagnosis of CF, their family or parents carry a lot of the burden. Therefore, we believe that non-medical treatments that positively affect mental health should also be extended to parents or families. In summary, we believe that non-medical treatment options have the potential to be a valuable additional therapy and can have a positive impact on the emotional well-being of CF patients. As there is not much data, we cannot specify the extent of the positive influence that non-medical therapies have on individuals with cystic fibrosis. However, we saw the urgency of prioritizing mental health, as ignoring it may negatively impact their lives. We suggest that further research in this field should be conducted with a more significant number of participants for a longer time.
